# Dentate Gyrus Circuitry Features Improve Performance of Sparse Approximation Algorithms

**DOI:** 10.1371/journal.pone.0117023

**Published:** 2015-01-30

**Authors:** Panagiotis C. Petrantonakis, Panayiota Poirazi

**Affiliations:** Institute of Molecular Biology and Biotechnology, Foundation for Research and Technology-Hellas, Heraklion, Greece; Martin Luther University, GERMANY

## Abstract

Memory-related activity in the Dentate Gyrus (DG) is characterized by sparsity. Memory representations are seen as activated neuronal populations of granule cells, the main encoding cells in DG, which are estimated to engage 2–4% of the total population. This sparsity is assumed to enhance the ability of DG to perform pattern separation, one of the most valuable contributions of DG during memory formation. In this work, we investigate how features of the DG such as its excitatory and inhibitory connectivity diagram can be used to develop theoretical algorithms performing Sparse Approximation, a widely used strategy in the Signal Processing field. Sparse approximation stands for the algorithmic identification of few components from a dictionary that approximate a certain signal. The ability of DG to achieve pattern separation by sparsifing its representations is exploited here to improve the performance of the state of the art sparse approximation algorithm “Iterative Soft Thresholding” (IST) by adding new algorithmic features inspired by the DG circuitry. Lateral inhibition of granule cells, either direct or indirect, via mossy cells, is shown to enhance the performance of the IST. Apart from revealing the potential of DG-inspired theoretical algorithms, this work presents new insights regarding the function of particular cell types in the pattern separation task of the DG.

## Introduction

The hippocampal formation is one of the most important information processing units in the brain, critically implicated in spatial [1], associational and episodic memory storage and retrieval [2]. The hippocampus is a cascade of different subregions dedicated to perform distinct and specific functional processes. Information enters from the Entorhinal Cortex (EC, the upstream area of hippocampus) and passes through the Dentate Gyrus, the CA3, CA2 and CA1 regions, where it is processed accordingly to form neuronal representations of memories that will be stored and/or retrieved. Finally, suitably manipulated information from the hippocampus is projected back to the cortex for further processing.

**Figure 1:**
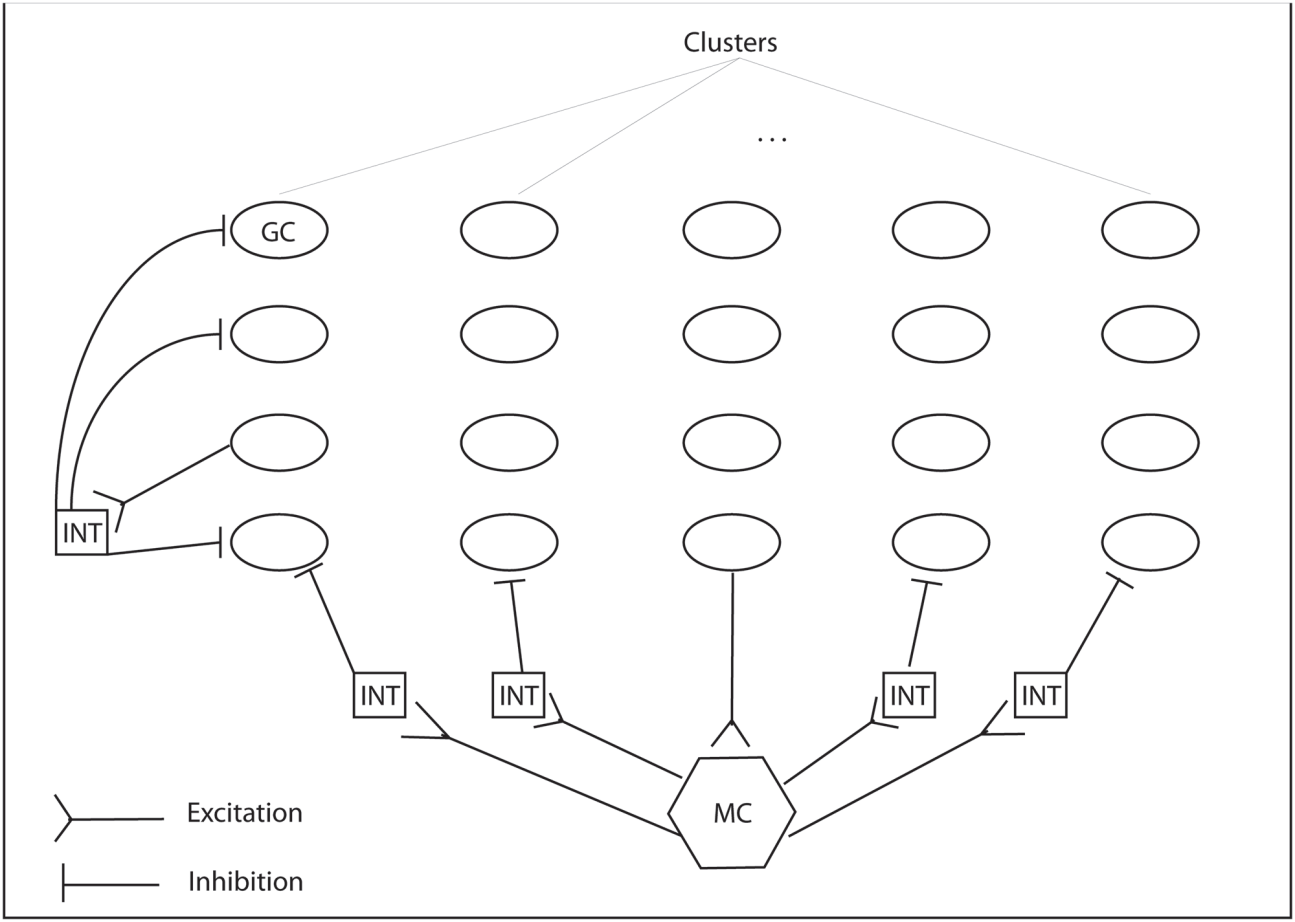
Intra- and inter- cluster inhibition in Dentate Gyrus. In intra-cluster inhibition(first column of GCs), the most excited GC excites an interneuron which projects back to inhibit other GC within the same cluster. The same mechanism holds for the inter-cluster inhibition mediated by MCs. (MC: Mossy Cells, GC: Granule Cells, INT: Interneurons).

The first information “processor” (subregion) in the hippocampal cascade is the Dentate Gyrus (DG). The main encoding cells of DG are Granule Cells (GC) that receive sensory information from EC and detonate the CA3 area (the downstream hippocampal subregion) by strong synapses that are formed via mossy fiber axons [3]. These principal cells are packed within the Granule Cell Layer [4] of the DG, and have been proposed to be organized in different clusters [5]. Except for principal granule cells, DG incorporates other excitatory cells, called Mossy Cells (MC), and various inhibitory cells [6], with pyramidal Basket Cells (BC) being the most important and intensively studied [4]. Both MCs and BCs are located in another layer of DG, the Polymorphic Cells layer, and receive excitatory afferents from principal GCs. The BCs project back to GCs layer and inhibit cells within the same cluster [5] whereas MC exhibit more distributed excitatory connections to GCs. Apart from the excitation to GC, MCs also excite BCs and it is evidenced that the *net effect* of MCs excitation is to inhibit GC via BCs in an inter-cluster manner [7, 8]. Thus, both MCs (via the conjectured net inhibition effect) and BCs inhibit GCs by inter- and intra-cluster lateral inhibition, respectively. Fig. 1 illustrates the two types of lateral inhibition.

It has been proposed [2, 9–11] that the hippocampus performs two important computations, the so called pattern separation and pattern completion tasks. Pattern separation refers to the ability of the network to reduce or eliminate the overlap between similar inputs, before they are further processed and stored in downstream areas, in order to reduce interference during memory recall. Pattern completion concerns the ability of the network to retrieve stored memory patterns when triggered with partial or noisy inputs. In many models of the hippocampus, the DG region is considered as a preprocessing unit that performs pattern separation on EC inputs. This conjecture is supported by anatomical and network features such as the sparsity of neuronal activation patterns and the existence of detonator synapses between DG and the downstream CA3 area [12]. Specifically, sparsity allows for inputs currying similar information to be encoded into non-overlapping GC populations [13]. This sparse code is subsequently imposed on CA3 pyramidal neurons via the strong mossy fiber connections, triggering the storage of new memories [14]. The emergence of sparse representations in the DG has been attributed to the interplay of intra- and inter- cluster inhibition [5, 15]. According to this model GCs activate MCs and BCs and the subsequent net inhibition constrains DG firing, allowing for sparse representations (see Fig. 1). This mechanism enables DG to translate the noisy and dense signal of the upstream cortical areas into a sparse and specific code to be further manipulated by the hippocampal formation for the efficient storage and recall of multiple memory items [16].

Code transformations, from redundant signals to sparse representations, like the ones proposed to be performed in the hippocampus, are extensively studied in the Signal Processing field. For instance, an *N*-dimensional signal, e.g., an image,*f*, could be decomposed into few components of a wavelet-based dictionary Ψ, i.e., *f* = Ψ*x*, where *x* is sparse, i.e., it has very few non-zero elements. It was recently proved analytically [17] that once you have few random measurements *y* (with dimension *M* < *N*) of the initial signal, e.g., *y* = Φ*f*, it is possible to uniquely identify *x* as long as it is sufficiently sparse. In many cases one can exactly recover such a sparse signal *x* as the solution to:

$$\min{\left\|x\right\|}_{1}\;s.\;t.\;\;y=Ax$$ (1)

where ${\left\|x\right\|}_{1}$ denotes the *l*_1_ norm, i.e., the absolute sum of the components of vector *x*. It can be shown that the *l*_1_ norm is an adequate sparsity constraint instead of the *l*_0_ norm (the number of non-zero elements of a vector), which leads to computationally intractable algorithms. Finding algorithms that efficiently solve Eq. 1 is an area of great interest since matrix *A* and vector *x* can contain millions of entries [18], in which case traditional linear programming algorithms are too slow. Given the importance of the sparsity constraint in these algorithms and the efficiency of the DG to produce sparse representations it would be interesting to investigate the role of inter- and intra- cluster inhibition in such algorithms and, vice versa, extract valuable insights regarding the role of the implicated cell types in pattern separation as a code transformation task.

One prevalent family of such algorithms are the iterative thresholding algorithms [19]. It has been found both theoretically and empirically that a sparsity-promoting process introduced by the thresholding procedure can solve *l*_1_ minimization problems, such as the one in Eq. 1, provided that they have sufficiently sparse solutions. Starting from *x*_1_ = 0, the iterative rule applies as:

$$x_{i+1}=\eta \;\left(x_{i}+\kappa \;\cdot \left(A^{T}\left(y-Ax_{i}\right)\right),\;t\right)$$ (2)

where *η*(.) is the thresholding function, *t* the threshold, and 0 < κ < 1 is a relaxation parameter. The soft thresholding function $\eta_{s}\left(x\right)=\mathrm{sgn}\left(x\right){\left(\left|x\right|-t\right)}_{+}$ with a fixed threshold *t* is directly related with the *l*_1_ norm (see S1 Text) and has been used in various settings (see for example [20]) and extensively analyzed with regard to its convergence [21].

In this work we investigate whether incorporation of the DG inhibitory mechanisms can improve the performance of the Iterative Soft Thresholding (IST) algorithm by enhancing its sparsification function. Particularly, we investigate the performance of the IST-based, sparse approximation task (Eq. 1), whereby the *x* vector is considered to be approximated by the population activity of the DG in terms of the GCs firing rates. Thus, we examine only the case of positive values approximation.

In the subsequent paragraphs we evaluate the results of the new sparse approximation algorithm that incorporates the two sources of potential inhibition in the DG, hereby termed DG-IST algorithm. Moreover we try to infer the biophysical mechanisms (i.e. cell-type specific connections) that could account for the improved performance of the DG-IST algorithm. We investigate whether such mechanisms can be utilized to implement new Winner-Take-All approaches that select which cells (*x* vector elements) fire (change) during the iterative approximation process [22]. Apart from the implications of our method with respect to algorithmic improvements in the signal processing field, we analyze the functional role of each inhibitory component on sparse approximation and infer the role of the corresponding cells in a hypothesized sparse approximation functionality of the DG in terms of the pattern separation task.

## Results

### Implementation of the DG-IST algorithm

Fig. 1 is a graphical illustration of the proposed algorithm, incorporating the simple inter- and intra- cluster inhibition. The organization of GCs is adopted from the work of Myers and Scharfman [5], where it is supposed to be structured in non-overlapping clusters. Each interneuron (INT) is activated by all (not shown in the Figure) GCs in a cluster and, in turn, it projects back to inhibit GCs of the very same cluster, implementing a form of ‘‘winner-take-all’’ competition. Thus we assume that, all, but the most strongly activated GCs within a cluster, receive inhibition [5]. While it is unlikely that the strongest GCs lose inhibitory connections entirely, this simplification reflects the realistic scenario where highly excitable GCs are able to overcome inhibitory inputs. The same mechanism for inter-cluster inhibition is evidenced to be implicitly mediated by MCs via disynaptic inhibition [7], as shown in Fig. 1.

In order to transfer the aforementioned inhibition mechanisms to the iterative IST algorithm, vector *x* is transformed into a matrix *x^m^*, where each column corresponds to a cluster of GCs. Specifically, the N-dimensional (N = 1000) vector *x* corresponding to the GC population, is divided into 25 non-overlapping clusters (matrix columns) each containing 40 elements [5]. In every iteration of the DG-IST algorithm, all but the most excited GCs (largest elements) in each column and each row are inhibited by subtracting suitably constructed matrices (see Materials and Methods), *I_s_* and *M_s_*, corresponding to intra- and inter- cluster inhibition, respectively (without loss of generality we consider inter-cluster inhibition to be imposed row-wise on matrix *x^m^*). Hence, the initial IST algorithm shown in Eq. 2 is hereby altered according to the equation:

$$x_{i+1}^{m}=\eta_{s}\;{\left(x_{i}^{m}+\kappa \;\cdot \left[{\left(A^{T}\left(y-Ax_{i}\right)\right)}^{m}-I_{s}-M_{s}\right],\;t\right)}^{}$$ (3)

where the *m* notation indicates that we refer to the matrix version of the corresponding vector as previously described. The soft thresholding function is applied to each element of the vector-matrix separately (see S1 Text for selected threshold value *t* and relaxation parameter *κ*).

In order to evaluate the performance of the DG-IST algorithm, the *x* vectors where generated with a sparsity degree*a a* = 2%. That is, only 2% (randomly selected) of the elements of vector *x* had non-zero values that were uniformly distributed in the interval [0,1]. The adopted 2% sparsity degree was based on experimental evidence reporting that sparse representations in DG consist of approximately 2–4% of the GCs population [23]. The vector *y* was subsequently estimated by the formula *y* = *Ax* (see Eq. 1) where matrix *A* is a random, Bernoulli, *M* × *N*, matrix with *M* ≥ *a* log(*N*/*a*) (*a* here is used in absolute values instead of percentage) [24] (see S1 Text for *M* estimation). The performance of both IST and DG-IST algorithms was then evaluated on the task of approximating the initial vectors *x*, given *y* and *A*.

### Case specific evaluation of the DG-IST algorithm

**Figure 2:**
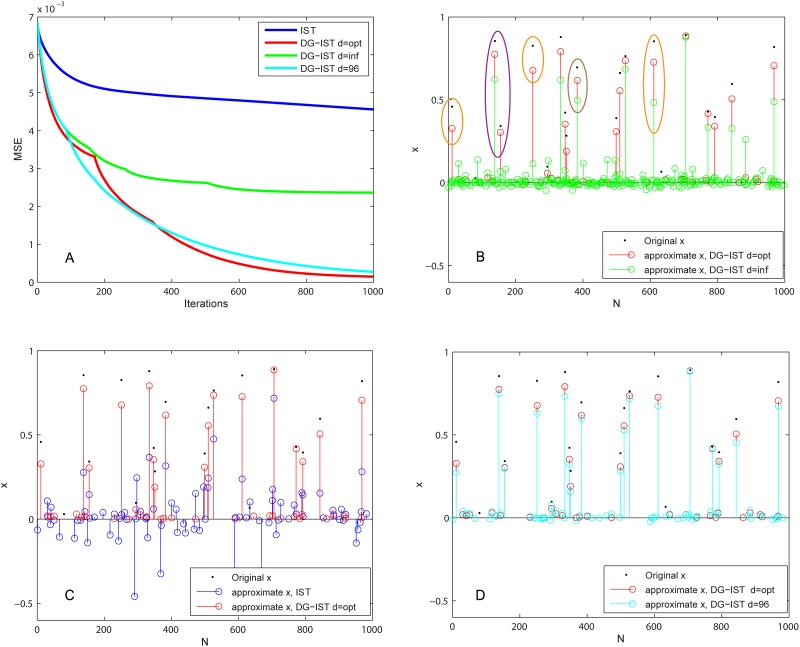
DG-IST performance. (A) MSE vs. Iterations for IST (blue), DG-IST with d = inf (green) DG-IST with d = opt = 171 (red), and DG-IST with d = 96 (cyan). (B) Sparse approximation of vector *x* by DG-IST with d = opt = 171 (red) and DG-IST with d = inf (green). Purple and orange highlighted stems correspond to elements within the same column and row of matrix *x^m^*, respectively. Brown highlighted stem corresponds to a non-zero element that belongs to a row and a column with no other non-zero elements. (C) Sparse approximation of vector *x* by DG-IST with d = opt = 171 (red) and IST (blue). (D) Sparse approximation of vector *x* by DG-IST with d = opt = 171 (red) and d = 96 (cyan).

Fig. 2A shows the performance of the simple IST algorithm (blue) for *T* = 1000 iterations for a randomly selected vector *x*. The vertical axis denotes the Mean Squared Error, given by $MSE=\frac{1}{N}\sum_{k=1}^{N}{\left(x_{k}-{\hat{x}}_{k}\right)}^{2}$. The DG-IST performance is depicted in green (cyan and red lines illustrate the performance of other versions of the DG-IST that will be described below). As seen in the figure, the MSE reduction rate slows down dramatically after *T* ≈ 200 iterations, and seems to saturate after *T* ≈ 500 (Fig. 2A, green line). This saturation is attributed to the fact that each cluster (column of *x^m^*) or group of GCs affected by inter-cluster inhibition (row of *x^m^*) can contain more than one non-zero elements, i.e., elements to be approximated. The “winner-take-all” mechanisms of the inter- and intra- cluster inhibition, however, allow only the largest elements within a column or a row of the *x^m^* matrix to be approximated, disregarding other non-zero elements. As a result, the MSE reaches a plateau reflecting the algorithm’s failure to approximate these additional non-zero elements. This problem can be overcome by decreasing the number of GCs receiving inhibition at the sight of saturation, thus, allowing approximation of subsequent non-zero *x* elements within a column or row. This artificial decay of inhibition is implemented by modifying matrices *I*_s_ and *M*_s_, every *d* iterations, such that all, *but* the two, three etc. most strongly activated GCs (*x* elements) within a column or row receive inhibition.

The performance of the DG-IST without decay of inhibition (*d* = inf), is illustrated in Fig. 2A in green, as previously reported. Values of *d* = 1,…,500 for that particular *x* vector were explored in order to find the optimum *d* value. The performance of DG-IST with optimum *d* = opt = 171 is shown in Fig. 2A in red. Note the substantial decrease in the MSE after *T* = 1000 iterations in comparison with the previous version of DG-IST, where *d* = inf (i.e., without decay of inhibition).

The approximation accuracy of DG-IST for *d* = inf and *d* = *opt* = 171 is shown in Fig. 2B. Black dots denote the original *x* vector while green and red stems illustrate the corresponding approximation by the DG-IST algorithm with *d* = inf and *d* = 171, respectively. Purple and orange cycles show *x* elements that reside in the same column and row of *x^m^*, respectively. Note that for a given column (purple cycle), when *d* = inf only one (of the two *x* elements) is approximated (green stems) during the iteration process whereas the other is suppressed by intra-cluster inhibition. The same can be seen for multiple elements within a row (orange circles, green stems). This problem is resolved when *d* = *opt* = 171, where the gradual decrease of inhibition allows for better approximation of multiple *x* elements (red stems). The same comparison between the original IST algorithm and the DG-IST, with *d* = *opt* is shown in Fig. 2C.

While the gradual decrease of inhibition (*d* ≠ inf) is vital for the efficiency of the proposed DG-IST algorithm, the *d* value that determines the iteration step at which removal of inhibitory inputs takes place is case-specific. For instance, approximation of different *x* vectors requires different *d* optimum values. In order to investigate if there is a global *d* value for a certain size, *N*, and sparsity degree of the vector *x*, 100 different vectors, with the same size and sparsity properties were constructed and the MSE error curve after 1000 iterations was calculated for *d* = 1,…,500. S1 Fig. illustrates the average curve of the aforementioned 100 cases, that exhibits its minimum at *d* = 96. Fig. 2A (cyan line) shows the performance of DG-IST with this minimum value of *d* = 96 and demonstrates the slight difference between the DG-IST with the optimum *d* value and *d* = 96. This negligible difference is also illustrated with respect to the approximation of each *x* element in Fig. 2D. Thus, for a specified sparsity level and a given problem dimensionality (*N*), it is possible to extract a global *d* value that allows efficient approximation of any given instance of vector *x*. Nevertheless, the determination of a general optimum *d* value that is independent of dimensionality and sparsity constraints should be further investigated and relative considerations are described in the “Discussion” section.

### Functional interpretation of the DG-IST algorithm

**Figure 3:**
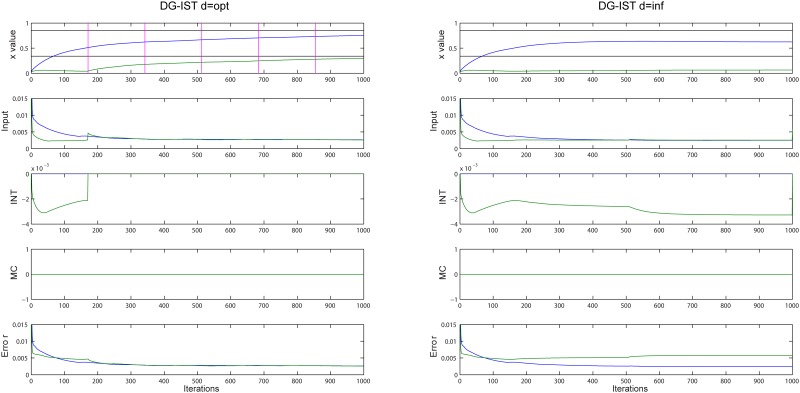
Evolution of approximation for purple-highlighted elements in Fig. 2B, using DG-IST with d = opt = 171 (left panel) and DG-IST with d = inf (right panel). First row of each panel shows the approximation evolution of the two elements, black horizontal lines declare the original elements to be approximated and vertical pink lines (only left panel) show the iterations at which elimination of inhibition takes place for the second largest element in the corresponding column of *x^m^*. The second row of each panel illustrates the Input to each GC through the iterative process, $input=\kappa \;\cdot \left[{\left(A^{T}\left(y-Ax_{i}^{m}\right)\right)}^{m}-I_{s}-M_{s}\right]=\kappa \cdot (Error+INT+MC)$. The Error, MC, and INT values that add up to form the Input value are shown in the remaining rows of each panel.

In order to understand the functional aspects of the proposed DG-IST algorithm, it is necessary to investigate the way *x* elements within the same, e.g., cluster (column of matrix *x^m^*), are approximated. The evolution of the approximation of *x* elements within the purple cycle in Fig. 2B (same cluster-column) can be seen in Fig. 3, for the DG-IST with *d* = *opt* = 171 and DG-IST with *d* = inf in the left and right panels, respectively. The first row of each panel (left and right) shows how the approximation of the two elements evolves: black horizontal lines show the original *x* elements to be approximated and vertical pink lines (in the left panel only) show the iterations at which elimination of inhibition takes place for the second (*T* = *d* = 171), third (*T* = 2*d* = 342) etc., largest elements in the corresponding column of matrix *x^m^*. The second row of each panel illustrates the input to each GC (*x* element) through the iterative process, i.e., $input=\kappa \;\cdot \left[{\left(A^{T}\left(y-Ax_{i}^{m}\right)\right)}^{m}-I_{s}-M_{s}\right]=\kappa \cdot (Error+INT+MC)$ (see Eq. 3). The INT, MC, and Error values that add up to form the Input value are shown in the remaining rows of each panel, respectively. It is profound that without artificially eliminating inhibition, the second largest element remains very low (first row, right panel, green line), due to the subtraction of a constant value originating from the intra-cluster inhibition mechanism (third row, right panel, green line). On the other hand the same element is adequately approximated when gradual elimination of inhibition is used (first row, left panel, green line). Specifically when *T* = *d* = 171, inhibition from INT (third row, left panel, green line) is eliminated and thus a severe discontinuity in the Input value (second row, left panel, green line) allows for the DG-IST algorithm to proceed with the approximation of the second largest element. Similarly, S2 Fig depicts the evolution of the elements highlighted by the orange cycles in Fig. 2B, which belong to the same row of matrix *x^m^*, where the same phenomenon is illustrated for the second and third largest elements.

Interestingly, the gradual removal of inhibition also enhances the approximation of *x* elements that have no other elements to compete with in the same column/row, as is the case highlighted with the brown cycle in Fig. 2B. Despite the fact that this particular element is from the beginning dominant within its column and row, the alteration of the Error variable due to the elimination of inhibition in other columns (GCs clusters) and rows also affects the Error element for this particular value. This causes a slightly slower decrease in the Error term and, as a result, the evolution of the approximation of that element is affected due to the soft thresholding function (see S3 Fig).

**Figure 4:**
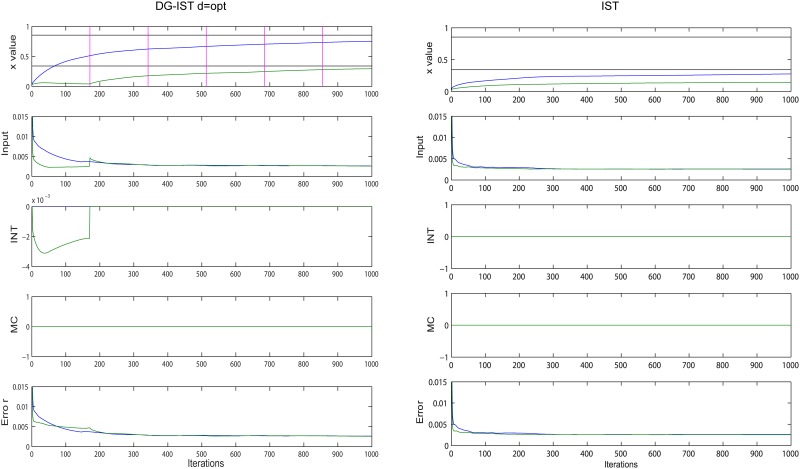
Evolution of approximation for purple-highlighted elements in Fig. 2B, using DG-IST with d = opt = 171 (left panel) and IST (right panel). First row of each panel shows the evolution of the two elements, black horizontal lines declare the original elements to be approximated and vertical pink lines (only left panel) show the iterations at which elimination of inhibition takes place for the second largest element in the corresponding column of matrix *x^m^*. The second row of each panel illustrates the Input to each GC through the iterative process, $input=\kappa \;\cdot \left[{\left(A^{T}\left(y-Ax_{i}^{m}\right)\right)}^{m}-I_{s}-M_{s}\right]=\kappa \cdot (Error+INT+MC)$. The Error, MC, and INT values that add up to form the Input value are shown in the remaining rows of each panel.

A similar comparison, this time between IST and DG-IST with *d* = *opt*, is shown in Fig. 4, where the approximation history for the elements in the purple cycle (same cluster) of Fig. 2B is depicted. Note that in the IST algorithm there are no MC and INT components. The corresponding graphs are depicted here for consistency reasons. The dominant difference between the two algorithms is that IST approximates the two elements simultaneously, and it essentially fails to approximate both of them, whereas the DG-IST initially isolates the most dominant element while keeping constant the second largest element until the first inhibition elimination, i.e., until *T* = *d* = *opt*. This is accomplished through the inhibition mechanism; in this case, by the intra-cluster inhibition (this is the reason that MC component is zero). As a result, the Error term of the most dominant element (blue line) decreases slower in DG-IST than in the IST algorithm and the soft thresholding function leads to a faster increase of the element under approximation. As soon as the elimination of inhibition happens, a slight jump in the Input value triggers the approximation of the second dominant element within the particular cluster. It should also be noted that the Error component of the second largest element (green) remains nearly *constant* (and so does the corresponding element under approximation—green line, first row) until the first elimination, which then enables the approximation of this particular element. In contrast, in the IST algorithm the Error term decreases rapidly. Taking into account that the Error term is the one subject to optimization as the task proceeds (see Eq. 1), it is vital that this parameter is dissociated for the two (or more) elements under consideration. This is the most valuable contribution of the proposed algorithm: *it dissociates the approximation process for multiple elements within the same column and/or row of the x^m^ matrix (i.e. for multiple highly active GC cells) and this is accomplished by the intra- or inter-cluster inhibition that preserves the Error term for the less dominant x elements (i.e. 2^nd^, 3^rd^ etc highly active GC cells) until inhibition elimination takes place*. As can be seen in Fig. 3 (right panel), this dissociation is not exploited unless the gradual elimination of inhibition takes place.

**Figure 5:**
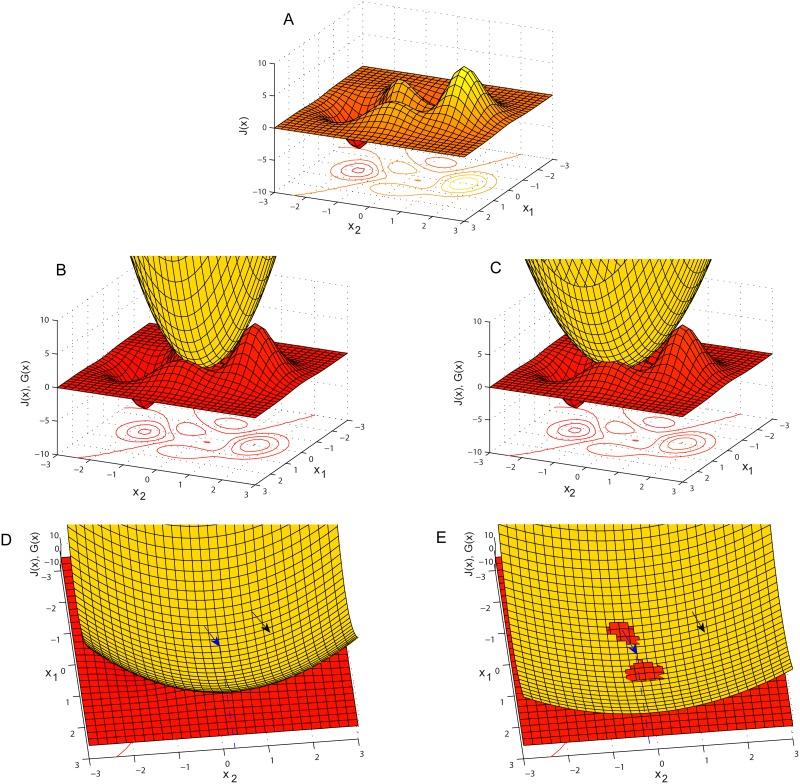
Majorization-Minimization by IST and DG-IST. (A) the *J*(*x*) function to be minimized. (B) The *G*(*x*) (yellow surface) function where *G*(*x*) ≥ *J*(*x*) ∀ *x* and *G*(*x_k_*) = *J*(*x_k_*), IST algorithm. (C) *G*(*x*) ≥ *J*(*x*) ∀ *x_2_* with *x*_1_ = *const* and *G*(*x_k_*) = *J*(*x_k_*), DG-IST algorithm. (D) *x_k_* is the initialization point of vector *x* (black arrow) and blue arrow indicates the $x^{\prime }=\left({x^{\prime }}_{1},\;{x^{\prime }}_{2}\right)$ where *G*(*x*) is minimized, IST algorithm. (E) *x_k_* is the initialization point of vector *x* (black arrow, same as in (D)) and blue arrow indicates the $x^{\prime \prime }=\left({x^{\prime \prime }}_{1},\;{x^{\prime \prime }}_{2}\right)$ where *G*(*x*) is minimized, DG-IST algorithm. Notice that, ${x^{\prime \prime }}_{2}<{x^{\prime }}_{2}$, thus closer to the point where *J*(*x*) is minimized (see (A)).

The mathematical interpretation of the abovementioned dissociation of, e.g., elements *x*_1_ and *x*_2_, can be explained if we take into account that the IST algorithm is a Majorization-Minimization (MM) procedure [25] (see S1 Text). In order to simply illustrate the difference of the approximation process between the two algorithms, assume that we want to find the two elements *x*_1_ and *x*_2_ that minimize the function *J*(*x*) of Fig. 5A. According to the MM process, if it is difficult to minimize function *J*(*x*), another function, *G*(*x*) is minimized for which, *G*(*x*) ≥ *J*(*x*) ∀ *x* and *G*(*x_k_*) = *J*(*x_k_*) (Fig. 5B, yellow surface), where *x_k_* is the initialization point for vector *x* (Fig. 5D, black arrow). As soon as the vector *x*′, that minimizes *G*(*x*), is found (blue arrow in Fig. 5D), then *x_k_* ← *x*′ and the MM process continues with a new *G*(*x*). The DG-IST algorithm, chooses a different *G*(*x*) (see Fig. 5C) such that, *G*(*x*) ≥ *J*(*x*) ∀ *x_2_* with *x*_1_ = *const* (this value represents actually the second dominant element to be approximated, see Fig. 3, left panel, first row, green line) and *G*(*x_k_*) = *J*(*x_k_*). For instance, in Fig. 5E, a different *G*(*x*) is chosen but the optimization process starts from the same *x_k_* (black arrow). The DG-IST algorithm finds the minimum of *G*(*x*) with constant *x*_1_ by changing *x*_2_ (in Fig. 3: constant green line in left panel while most dominant value (blue line) is under approximation, i.e., changes). Thus faster approximation of *x*_2_ is accomplished in comparison with the IST algorithm (see blue dashed lines in Fig. 5D and 5E. In Fig. 5E, the approximated *x*_2_ is closest to the global minimum of *J*(*x*)). As soon as the elimination of the inhibition for element *x*_1_ happens, the whole process resembles the one of the simple IST algorithm for the particular cluster where *x*_1_ and *x*_2_ elements reside. Finally, the same mechanism applies for three or more elements within the same, e.g., column (cluster) of matrix *x^m^*.

### Case independent evaluation of the DG-IST algorithm

**Figure 6:**
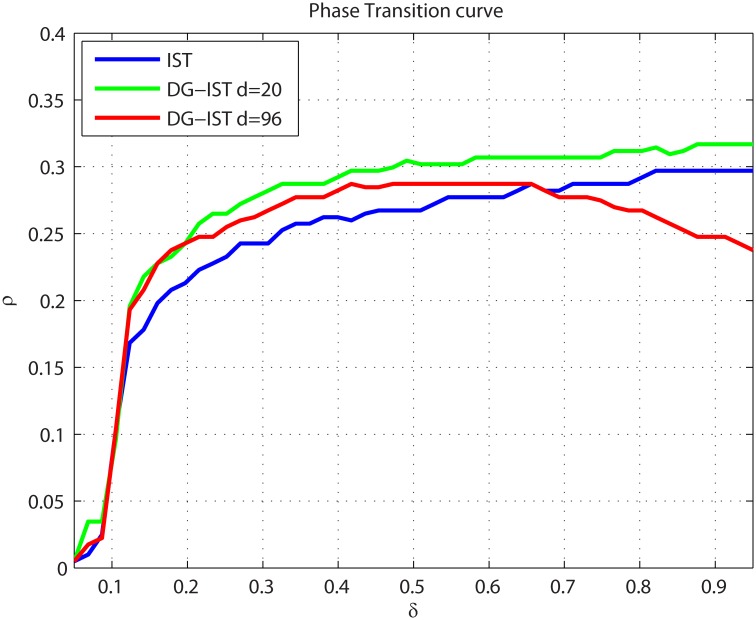
Phase transition curves for DG-IST with *d* = 96 (red line), *d* = 20 (green line) and IST (blue line).

The experiments described so far evaluated the performance of the new DG-IST algorithm in a case-specific manner. In order to evaluate the algorithm independently of the *x* vector, the sparsity-undersampling tradeoff must be tested by estimating the phase transition (PT) curve [26]. Fig. 6 illustrates the PT of IST (blue) and DG-IST (red) with *d* = 96. The domain of (δ, *ρ*) = (*M*/*N*,*a/M*) ∈ (0,1) is divided in two phases: the “success” phase and the “failure” phase. The former refers to the case where sparse approximation is successful in terms of a predefined target (See Materials and Methods) and the latter refers to the failure of the sparse approximation process. The region above the PT curve represents the “failure” case whereas the region below it represents the “success” case. Thus, better performance is depicted as a larger lower region compared to the upper one. As declared from its definition, $\delta =\frac{Μ}{Ν}$, is the undersampling fraction, i.e., how many measurements of a signal *f* are used for the approximation, in relation to the size of that signal. Furthermore, $\rho =\frac{\alpha }{M}$, is a measure of the sparsity of the signal *x*. According to Fig. 6, DG-IST outperforms the simple IST algorithm, except for the cases where, approximately, *δ* ≥ 0.7. For large *δ* values, the sparsity degree *α* = *ρ M* rises accordingly and, as a result, the probability of having many non-zero elements within a single column or row of matrix *x^m^* rises as well. The PT curve of DG-IST (red) in Fig. 6 is estimated with *d* = 96. Note that as the signal becomes denser, i.e. less sparse (i.e., *δ* ≥ 0.7), faster elimination of inhibition (smaller *d*) is necessary in order to approximate less dominant elements within, e.g., a cluster. Moreover, for *d* = 96, at most 10 elements can be relieved from inhibition after *T* = 1000 iterations whereas, theoretically, it is possible to have 40 non-zero elements within a cluster as the *x^m^* matrix in our simulations is a 40 × 25 matrix. In order to overcome this issue, the PT transition curve was re-estimated for DG-IST with much faster elimination of inhibition, using *d* = 20 (Fig. 6, green). Thus, all possible inhibition eliminations were accomplished until *T* = 800. In this case, DG-IST outperforms the IST algorithm for all undersampling fractions *δ* and, more importantly, with higher “success” than in the case of *d* = 96.

**Figure 7:**
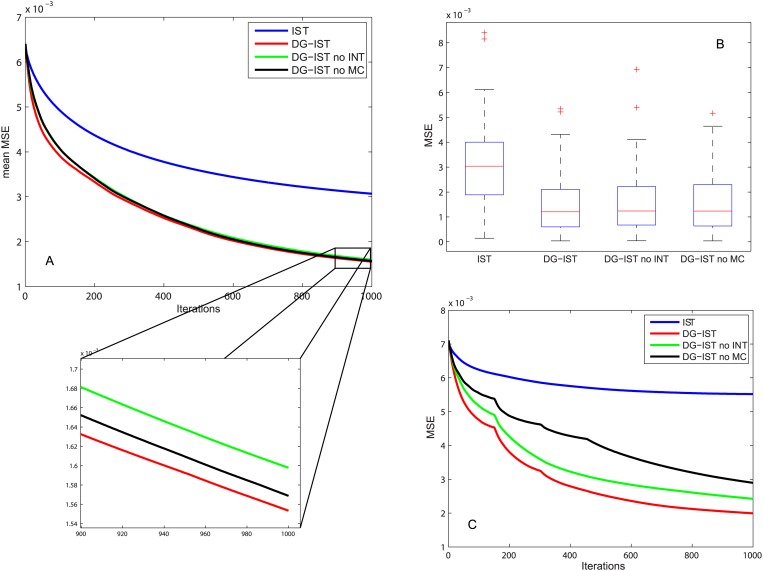
DG-IST without INT- or MC- mediated inhibition. (A) Mean MSE of 100 instances of *x* vectors with: *N* = 1000, *a* = 2%, *M* ≥ *a* ⋅ log(*N*/*a*) estimated using IST (blue), DG-IST with *d* = 96 (red), DG-IST with *d* = 96 without INT-mediated inhibition (green), and DG-IST with *d* = 96 without MC-mediated inhibition (black) (Inset: magnification of the last 100 iterations). (B) Boxplots of the MSE of 100 instances of *x* vectors with: *N* = 1000, *a* = 2%, *M* ≥ *a* ⋅ log(*N*/*a*) estimated using IST, DG-IST with *d* = 96, DG-IST with *d* = 96 without INT-mediated inhibition, and DG-IST with *d* = 96 without MC-mediated inhibition. (C) MSE of a specific instance of vector *x* with: *N* = 1000, *a* = 2%, *M* ≥ *a* ⋅ log(*N*/*a*) estimated using IST (blue), DG-IST with *d* = 96 (red), DG-IST with *d* = 96 without INT-mediated inhibition (green), and DG-IST with *d* = 96 without MC-mediated inhibition (black).

Finally, the cases where intra- (INT) or inter- (MC) inhibition is not used (i.e. *I_s_* or *M_s_* are omitted, respectively), were also investigated for the 100 instances of *x* vectors previously described (*N = 1000, a* = 2%, *M* ≥ *a* log(*N*/*a*)). Fig. 7A shows the mean MSE of all 100 vectors *x* for *T* = 1000 iterations. Note that after 1000 iterations the MSE differences between DG-IST (with optimal *d* value for each case) and its alterations without MC- or INT- dependent inhibition are not significant (see magnification insert). This is also evidenced by the box plot of the MSEs for the different cases after 1000 iterations (Fig. 7B). There is no significant difference between the various versions of the DG-IST algorithm but only between DG-IST and simple IST. Nevertheless, the analysis presented here incorporated both MC and INT inhibition as there were cases like the one in Fig. 7C where both inhibition mechanisms played an important role in sparse approximation. Overall, these results suggest that the two types of inhibition are important, but one can often correct for the biases introduced by the elimination of the other, implying some form of redundancy.

## Discussion

This work uses features of the DG circuitry to extend and improve the state of the art IST algorithm which is extensively used for sparse approximation tasks. Like other approximation algorithms, the performance of the simple IST can be improved by optimizing its parameters, e.g., the threshold for the soft thresholding function *η_s_* [19]. This work has shown that incorporation of DG features is sufficient to improve performance of the simplest form of IST, without extensive parameter optimization. Moreover, as shown in Fig. 7, incorporation of either the intra- or inter- cluster inhibition would generally lead to similar results as in the case were both inhibitory mechanisms are used. Hence, the usage of only one inhibitory mechanism and, thus, the discretization into clusters or groups of inter-cluster neurons reveals the potential parallelization of the IST algorithm provided that lateral inhibition along with functional modules of gradual elimination of inhibition are used for each parallel module.

The Error term of the GC input (see Eq. 3) implies the need of a plausible biological substrate for such an error signaling mechanism. Taking into account models that describe self-organized representations in hippocampus we assume that the Error term is provided by a hippocampal region other than the DG. Particularly, except for the direct projection of DG to CA3 there is also a backprojection path from CA3 to DG [27, 28]. The role of this backprojection on pattern separation in the DG was previously theoretically investigated [15] revealing its contribution to sparsity promotion through inhibition. Based on this evidence, we propose that the Error term is produced in the CA3 region and is fed back to DG in an effort to find the sparsest population in DG, i.e., the vector *x*. Then, the algorithm evolves and the next step (each step is considered as the loop DG-CA3-DG) incorporates the sparser projection from DG to CA3 causing a new CA3 representation that is iteratively compared with the initial representation caused by the perforant path [29].

Gradual elimination of inhibition and the corresponding, case-specific, step value *d* are important elements for the improved performance of the DG-IST algorithm. The requirement for case-specific, optimal parameters is definitely a drawback of any sparse approximation algorithm, which should be applicable to different case scenarios independently of vector *x* size, sparsity level, and the undersampling parameter. Note that elimination of inhibition is necessary to allow approximation of more than one element within, e.g., a cluster (column of *x^m^*). Since the elements of matrix *x^m^* are considered to be the firing rates of GCs, in a winner-take-all scheme of inhibition, the most excited GCs do not allow other GCs to fire, i.e., increase their firing rate, through feedback inhibition (each GC excites an interneuron which projects back to inhibit all GCs within a cluster but the one that initially excited the interneuron). Thus, elimination of inhibition serves as a correction step to the winner-take-all scheme of inhibition that has been implemented, by allowing slightly less stimulated GCs to fire, i.e., *x* vector elements to be approximated. The biophysical substrate of this regulation of inhibition may reside in the synaptic plasticity mechanisms that have been documented for DG interneurons [30]. Moreover, a mechanism that explains which GC fire within an inhibition framework like the one implemented here would further elucidate the importance of the *d* value and provide valuable insights on its robust determination and, simultaneously for its possible biological substrate. Such a mechanism was recently proposed [22] and is highly related to gamma oscillations, a prevalent rhythm within DG and hippocampus in general [31].

According to [22], gamma oscillations arise through feedback inhibition [31] but in a winner-take-all framework, in many cases, there are more than one winners (as desired for a cluster-column of *x^m^* with multiple elements to be approximated). Thus, an alternative theory, the E%-max winner-take-all has been proposed, according to which in any given gamma cycle, principal cells fire only if their excitation level is within E% of the excitation level of the most excited cell [22]. For instance, consider two neurons, N1 and N2, with N1’s activity being slightly higher (within E%) than N2’s. N1 will fire first and will trigger an interneuron. Because N2’s activity is only slightly lower than N1’s, it will reach the firing threshold before inhibition is fed back by the triggered interneuron. Thus, the E%-max mechanism is highly related to the artificially imposed relaxation of inhibition adopted in this work. Further investigation of the aforementioned mechanism is likely to reveal a global rule for the determination of *d*. According to this mechanism, the optimal *d* will be such that if the DG-IST algorithm is segmented to gamma cycles, the number of elements of the *x^m^* matrix (per column or row) that remain active and, thus, eligible for approximation, depends on the balance between excitation and inhibition. Specifically, it is evidenced that, cycle-by-cycle, gamma oscillations exhibit variations on amplitude that reflect changes in synaptic excitation spanning an order of magnitude [32]. In turn, excitation is proportionally counterbalanced through inhibition. Thus, this interplay between excitation and inhibition, which depends on the amplitude of gamma oscillations, may influence the E% criterion and subsequently determine the time point that an increasing number of cells become activated (*d* value in the DG-IST algorithm). We assume that, the algorithmic step is defined by the reciprocal connection of DG and CA3 regions whereas the determination of the time point *d* is influenced by the gamma-based excitation-inhibition counterbalance. Gamma oscillatory dynamics that account for DG-CA3 coupling have already been described [33], making possible a potential functional synergism between these two regions for the sparse approximation task described in this paper. Finally, we predict that the theta-based regulation of inhibition in hippocampus [34] corresponds to the regulation of the approximation task in terms of the fulfillment of the stopping criteria of the approximation, i.e., theta oscillations determine the number of required iterations for the DG-IST algorithm. Further investigation of this issue will not only clarify the role of *d* in the approximation procedure but will also reveal its biological substrate, presuming that it is related with the E%-max mechanism and active population selection in DG.

Finally, the improved performance seen by dissociating the approximation of multiple elements within a cluster (multiple GC activities) sheds new light on the contribution of MC- and INT-mediated inhibition for the pattern separation task. Pattern separation guarantees that two separate inputs from EC, even slightly different from each other, are coded by two separate activation patterns in CA3 [35]. The dissociation of the approximation imposed by DG-IST may relate to the fact that slight differences in EC input can recruit new GCs, that were initially inactive. In a sparse approximation task, pattern separation refers to the fact that measurements, *y*_1_ and *y*_2_ of different signals, *f*_1_ and *f*_2_ are due to the different representations, *x*_1_and *x*_2_ (see S1 Text for more information on sparse representations of signals based on a dictionary set Ψ). Thus, it would be interesting to investigate whether DG performs pattern separation in terms of estimating a sparse representation of two slightly different sources of activation in the cortex, as recently proposed [29]. For instance, assuming that the EC input refers to a dictionary Ψ. This dictionary could be the activity of grid cells [36] which can be considered as periodic basis functions. It has been proposed that grid fields of different spacing, combine linearly to generate place fields in the hippocampus, and could, thus, comprise a respective dictionary for place cells. Slightly different activation sources in the cortex that have sparse representations in a grid-cell-dictionary may have significantly different representation in DG, assuming that GCs activation is the product of a sparse approximation process. This will also elucidate the advantages that inter- or intra- inhibition provides to sparse approximation algorithms as illustrated here for the DG-IST approach. Nevertheless, the idea that DG performs pattern separation as an alternative manifestation of a sparse approximation task demands further experimental and computational investigation.

In sum this work shows that there are certain features in the DG that can account for the requirements imposed by an optimization algorithm such as the IST and can significantly improve its performance. These findings suggest that DG may play a key role in both sparse approximation and pattern separation functions, much like the two sides of the same coin.

## Materials and Methods

In this section we briefly describe the implementation of the algorithmic constituents of the DG-IST algorithm that were added to the original IST, i.e., the estimation of the *I_s_* and *M_s_* matrices and the gradual elimination of inhibition. We also give a comprehensive algorithmic description of the PT curve estimation.

### DG-IST algorithm

The main additions to the common IST algorithm was the *I_s_* and *M_s_* matrices, inspired by the intra- and inter- inhibition processes within DG. As illustrated in Fig. 1, GCs excite interneurons and MCs and then receive inhibitory feedback (explicitly or implicitly). In a winner-take-all scheme, only one neuron does not receive inhibition whereas that rest neurons within a cluster (or inter-cluster group of neurons) are inhibited. In the E%-max-winner-take-all scheme, more than one neurons, e.g., *r* neurons, may be active and not suppressed by the feedback inhibition. Thus, the *r* most excited GCs within a cluster or an inter-cluster group (i.e., within a column or row of matrix *x^m^*) are considered to surpass inhibition. Thus, in order to model this mechanism in a matrix-like and algorithmically efficient implementation, matrices *I_s_* and *M_s_* equal matrix *x^m^* except for the *r* largest elements of each row and column, respectively, which were substituted with zeros. More particularly, each column of matrix *I_s_* contains the same values of the corresponding column of matrix *x^m^*, except for the *r* largest values of that column which where substituted with zeros. The same row-wise estimation was implemented for matrix *M_s_* Finally, these matrices were subtracted from the initial *x^m^* matrix after being multiplied by the scaling factor *κ* (see Eq. 3).

The elimination-of-inhibition module is implemented by changing the *r* value. Thus, every *d* iteration steps we set *r* ← *r* +1, where initially *r* = 1.

### Phase Transition curve estimation

For each one of the two sparse approximation algorithms a specifically designed phase transition measurement experiment was conducted as follows [26]. A problem suite was defined, i.e., a matrix *A* and a vector *x* that comprise a problem instance (*A*,*x*). A grid of *δ* values is also defined in [0,1]. In particular, 50 equispaced values between 0.005 and 0.95 were used for *δ* grid construction. Subordinate to *δ* grid, another *ρ* grid is considered with 100 equispaced values between 0.01 and 0.99. For each (*δ, ρ*) ∈ [0,1]^2^, *F* problem instances are generated; here *F* = 20. In particular if the problem size, i.e., *N* is defined, we set *M* = ⌈ *δ·N* ⌉ and *α* = ⌈ *ρ·M* ⌉ and generate the aforementioned problem instances. The sparse approximation algorithms are called with the arguments (*y*,*A*) and lead to a solution, $\hat{x}$, which corresponds to a measure of success, declared as:

$$\frac{{\left\|y-A\hat{x}\right\|}_{2}}{{\left\|y\right\|}_{2}}\leq tol,$$ (4)

where *tol* = 10^−1^. The phase transition curve is defined as the value *ρ* at which success probability is 50%. We conduct this experiment 100 times and the median of the 100 individual curves are considered the final PT curve depicted in Fig. 6 for each algorithmic implementation.

## Supporting Information

S1 Fig Finding optimal *d* for certain *x* vector construction. 100 instances of vector *x* were generated with: *N = 1000, a* = 2%, *M* ≥ *a*·log(*N*/*a*). Blue line illustrates the mean MSE of these instances by DG-IST for *d* = 1,…,500. Minimum MSE value is at *d* = 96.
(TIF)

S2 FigEvolution of approximation for orange-highlighted elements in Fig. 2B, using DG-IST with d = opt = 171 (left panel) and DG-IST with d = inf (right panel).First row of each panel shows the evolution of the three elements, black horizontal lines declare the original elements to be approximated and vertical pink lines (only left panel) show the iterations at which elimination of inhibition takes place for the second and third largest elements in the corresponding row of matrix *x^m^*. The second row of each panel illustrates the Input to each GC through the iterative process, $input=\kappa \;\cdot \left[{\left(A^{T}\left(y-Ax_{i}^{m}\right)\right)}^{m}-I_{s}-M_{s}\right]=\kappa \cdot (Error+INT+MC)$. The Error, MC, and INT values that add up to form the Input value are shown in the remaining rows of each panel.(TIF)

S3 FigEvolution of approximation for brown-highlighted elements in Fig. 2B, using DG-IST with d = opt = 171 (left panel) and DG-IST d = inf (right panel).First row of each panel shows the approximation evolution for the element, black horizontal lines declare the original element to be approximated and vertical pink lines (only left panel) show the iterations at which elimination of inhibition takes place. The second row of each panel illustrates the Input to the GC through the iterative process, $input=\kappa \;\cdot \left[{\left(A^{T}\left(y-Ax_{i}^{m}\right)\right)}^{m}-I_{s}-M_{s}\right]=\kappa \cdot (Error+INT+MC)$. The Error, MC, and INT values that add up to form the Input value are shown in the remaining rows of each panel. Note, that for this case there are no MC and INT variables for the Input and the only parameter that changes is the Error. Magnifications show the difference in Error change between the two methods and the corresponding impact on the value approximation due to the soft thresholding process.(TIF)

S1 Text Iterative Soft Thresholding (IST) algorithm as a Majorization-Minimization (MM) optimization process and basic principles of Compressed Sensing theory. (PDF)
