# Supplementary figures and images for: Dentate Gyrus Circuitry Features Improve Performance of Sparse Approximation Algorithms
Source: PLoS One. 2015 Jan 30;10(1):e0117023. doi: 10.1371/journal.pone.0117023 (PMC4312091; doi:10.1371/journal.pone.0117023)

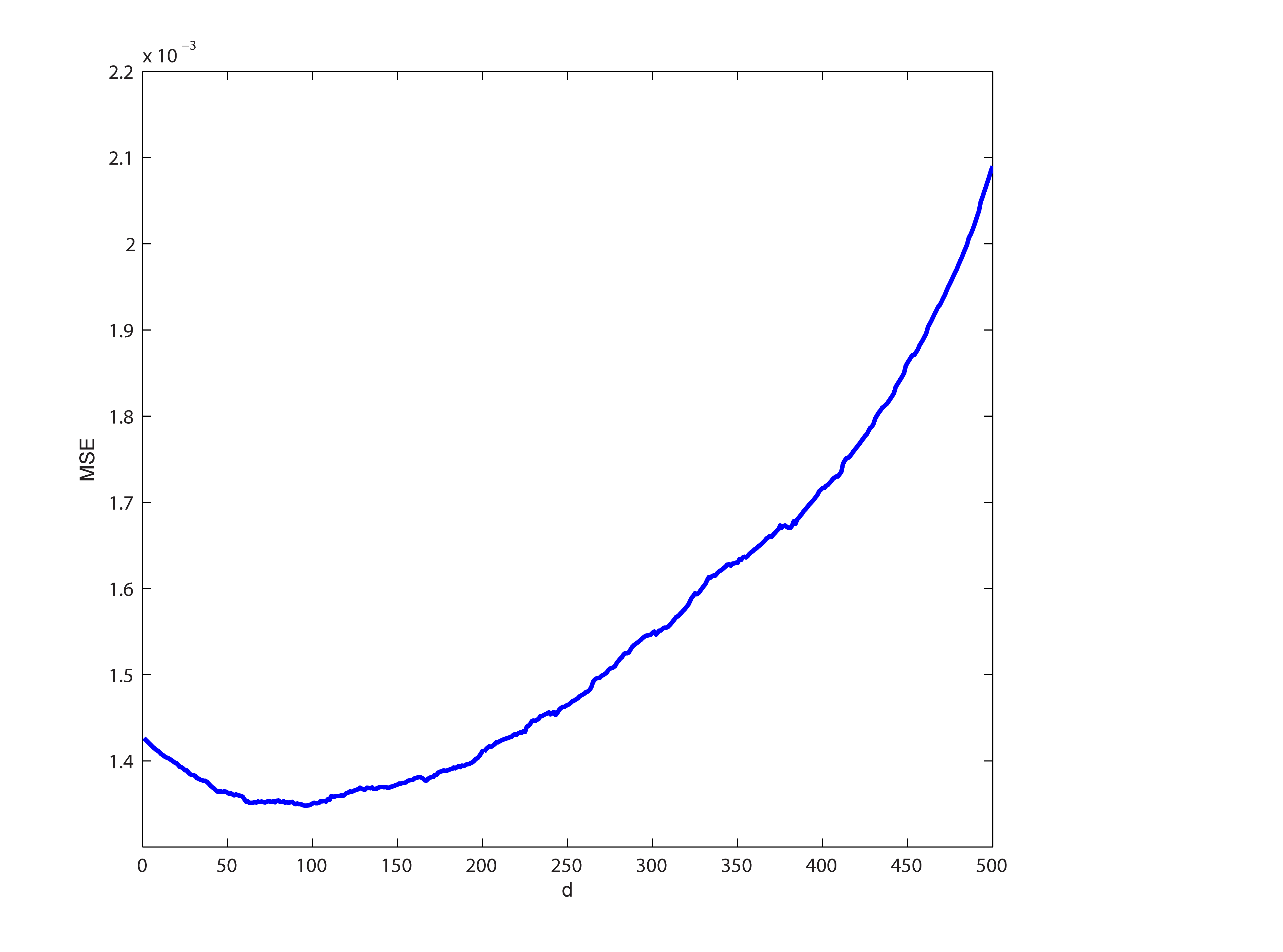

Supplement: S1 Fig — 100 instances of vector x were generated with: N = 1000, a = 2%, M ≥ a·log(N/a). Blue line illustrates the mean MSE of these instances by DG-IST for d = 1,…,500. Minimum MSE value is at d = 96. (TIF) [file pone.0117023.s001.tif]

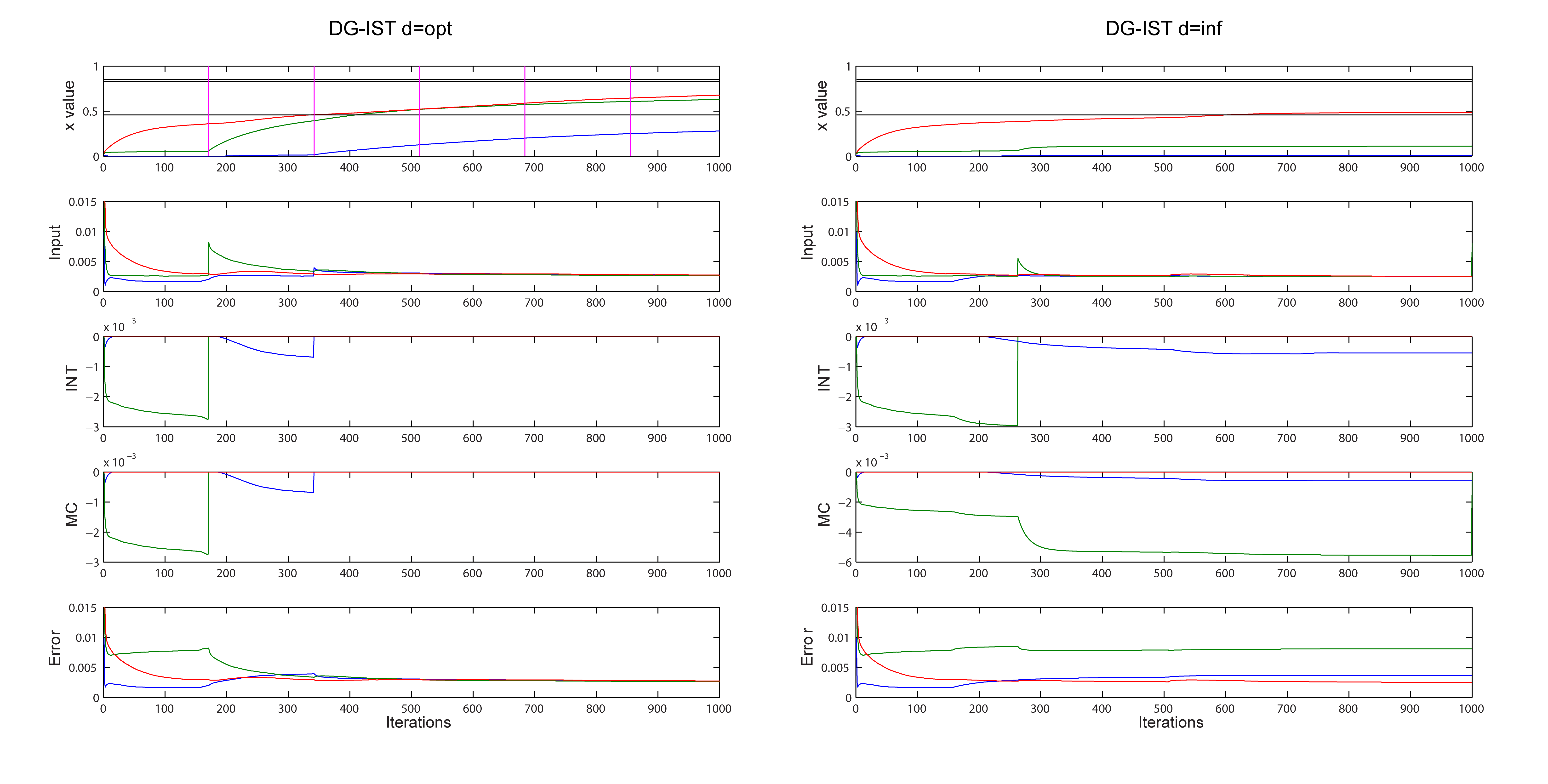

Supplement: S2 Fig — First row of each panel shows the evolution of the three elements, black horizontal lines declare the original elements to be approximated and vertical pink lines (only left panel) show the iterations at which elimination of inhibition takes place for the second and third largest elements in the corresponding row of matrix xm. The second row of each panel illustrates the Input to each GC through the iterative process, input=κ⋅[(AT(y−Axim))m−Is−Ms]=κ⋅(Error+INT+MC). The Error, MC, and INT values that add up to form the Input value are shown in the remaining rows of each panel. (TIF) [file pone.0117023.s002.tif]

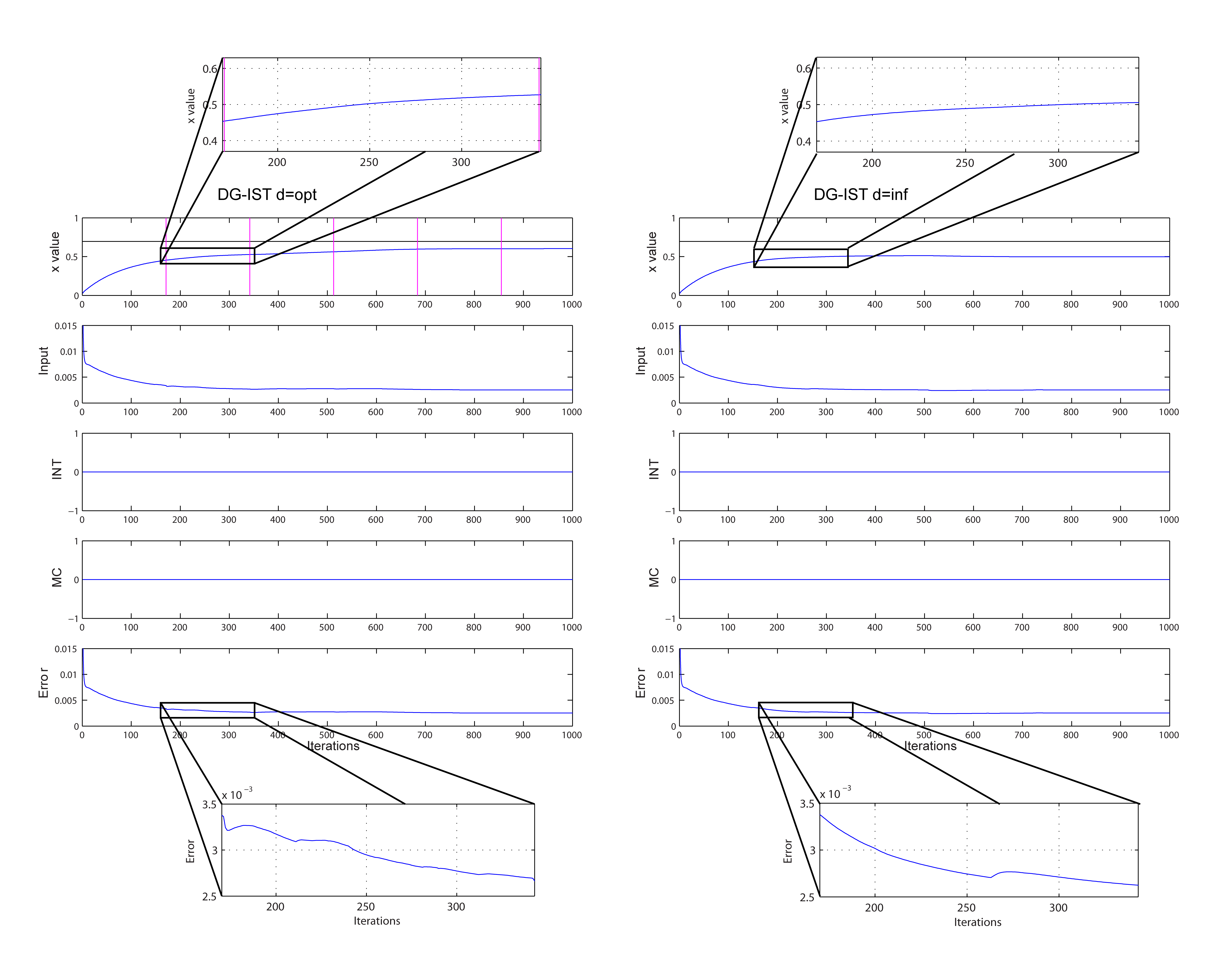

Supplement: S3 Fig — First row of each panel shows the approximation evolution for the element, black horizontal lines declare the original element to be approximated and vertical pink lines (only left panel) show the iterations at which elimination of inhibition takes place. The second row of each panel illustrates the Input to the GC through the iterative process, input=κ⋅[(AT(y−Axim))m−Is−Ms]=κ⋅(Error+INT+MC). The Error, MC, and INT values that add up to form the Input value are shown in the remaining rows of each panel. Note, that for this case there are no MC and INT variables for the Input and the only parameter that changes is the Error. Magnifications show the difference in Error change between the two methods and the corresponding impact on the value approximation due to the soft thresholding process. (TIF) [file pone.0117023.s003.tif]
