# Supplementary material for: Dentate Gyrus Circuitry Features Improve Performance of Sparse Approximation Algorithms
Source: PLoS One. 2015 Jan 30;10(1):e0117023. doi: 10.1371/journal.pone.0117023 (PMC4312091; doi:10.1371/journal.pone.0117023)
Supplement: S1 Text — (PDF) [file pone.0117023.s004.pdf]

Dentate Gyrus Circuitry Features Improve Performance of Sparse Approximation Algorithms

Panagiotis C. Petrantonakis and Panayiota Poirazi
Foundation for Research and Technology-Hellas (FORTH), Institute of Molecular Biology and Biotechnology, N. Plastira 100 GR-70013, Heraklion, Greece

Iterative Soft Thresholding algorithm

This section describes the derivation of the Iterative Soft Thresholding (IST) algorithm based on the Majorization-Minimization (MM) optimization process and on l_1 norm denoising [1].

The problem that IST deals with is the estimation of a vector x based on measurements y , with:

$$y = Ax \tag{S1}$$

where A is the matrix representing the measurement process. Hence, the approach is to estimate x from y by minimizing the function:

$$J(x) = \|y - Ax\|_2^2 + \lambda\|x\|_1 \ , \tag{S2}$$

where $\|.\|_n$ denotes the n-norm of the corresponding vector. In (S2), the first term represents the discrepancy between y and x and the second term is a penalty function. The λ parameter, is a positive value and determines the tradeoff between the two terms of equation S2.

The MM procedure [2] replaces a difficult to minimize problem to a sequence of simpler ones. Assuming that the minimization of the function $\mathrm{J(x)}$ is a difficult optimization problem, MM process defines a series of new functions, i.e., $\mathrm{G_k(x)}$, which majorize $\mathrm{J(x)}$ and tries to minimize them in order to conclude to the x that minimizes the original $\mathrm{J(x)}$. Thus, MM is an iterative process where, at each iteration, a vector $\mathrm{x_k}$ is found which ultimately converges to the minimum of function $\mathrm{J(x)}$. The iterative process is described as:

1. Set $k = 0$. Initialize $x_k = x_0$
2. Define $G_k(x)$ such that:

a. $G_k(x) \geq J(x) \; \forall \; x$

b. $G_k(x_k) = J(x_k)$

3. Find the minimizer of $G_k(x)$, i.e., x_{k+1}
4. Set $k \leftarrow k + 1$, go to step 2

Now, we will dissect the minimization problem by finding the minimizer of the first part of Eq. S2 and subsequently the minimizer of the second part. By combining the two solutions we will end up with the minimizer of Eq. S2.

If we want to minimize the first term of Eq. S2 we set :

$$J(x) = \|y - Ax\|_2^2 = (y - Ax)^t(y - Ax) = y^t y - 2y^t Ax + x^t A^t Ax \tag{S3}$$

By setting the derivative of S3 to zero we get:

$$\frac{\partial}{\partial x} J(x) = 0 \Rightarrow -2A^t y + 2A^t Ax = 0$$

Thus,

$$x = (A^t A)^{-1} A^t y \tag{S4}$$

Hence the minimization of S3 requires the solution of a system of linear equations. If the x vector is very large the computational burden of the solution may be huge. In order to avoid the solution of the system of equations we can use the MM process and define a new series of $G_k(x)$ that coincides with $J(x)$ at x_k but otherwise majorizes $J(x)$.

Thus, we define:

$$G_k(x) = \|y - Ax\|_2^2 + (x - x_k)^t (bI - A^t A)(x - x_k) \tag{S5}$$

which fulfills the constrains imposed by MM, as $G_k(x_k) = J(x_k)$ and the second part of S5 is non-negative as soon as b is greater or equal to the maximum eigenvalue of $A^t A$:

$$b \geq \max \quad eig(A^t A) \tag{S6}$$

securing that $G_k(x) \geq J(x) \; \forall \; x$. Now, in order to obtain x_{k+1} we need to minimize $G_k(x)$. Thus:

$$\frac{\partial}{\partial x} G_k(x) = 0 \Rightarrow$$

$$-2A^t y - 2(bI - A^t A)x_k + 2bx = 0 \Rightarrow$$

$$x = x_k + \frac{1}{a} A^t (y - Ax_k)$$

Hence, with the MM procedure, we obtain:

$$x_{k+1} = x_k + \kappa A^t(y - Ax_k), \kappa = \frac{1}{\alpha} \quad \text{(S7)}$$

Thus, Eq. S7 defines the procedure that minimizes the function $J(x)$ of Eq. S3.

In order to incorporate the l_1-norm part of the Eq. S2, we consider the minimization of a simpler form of Eq. S3, where $A = I$ but with the regularizing term of the l_1-norm. Thus:

$$J(x) = \|y - x\|_2^2 + \lambda\|x\|_1 \quad \text{(S8)}$$

It is not possible to differentiate $J(x)$ as the l_1 norm is not a differentiable function. Instead, we expand Eq. S8:

$$J(x) = (y_1 - x_1)^2 + \lambda|x_1| + \cdots + (y_N - x_N)^2 + \lambda|x_N| \quad \text{(S9)}$$

And we can minimize Eq. S9 by simply minimizing each individual part, i.e., by minimizing:

$$j(x) = (y - x)^2 + \lambda|x| \quad \text{(S10)}$$

Taking the derivative we have:

$$\frac{d}{dx} j(x) = 0 \Rightarrow$$

$$-2(y - x) + \lambda\, sign(x) = 0 \Rightarrow$$

$$y = x + \frac{\lambda}{2} sign(x) \quad \text{(S11)}$$

By solving Eq. S11 for x it is proved that the minimizer of $j(x)$ by applying the soft threshold rule to y with threshold $\frac{\lambda}{2}$. Thus the minimization of Eq. S8 is accomplished by:

$$x = \eta_s\left(y, \frac{\lambda}{2}\right) \quad \text{(S12)}$$

where

$$\eta_s(z, th) := sign(z) \max(0, |z| - th)$$

Finally, by combining the minimizers of Eq. S3 and S8 we end up with the minimizer of Eq. S2, i.e., the IST algorithm:

$$x_{k+1} = \eta_s(x_k + \kappa A^t(y - Ax_k), \frac{\lambda}{2b}) \quad \text{(S13)}$$

Where $\kappa = \frac{1}{b}$ and $b \geq \max \quad eig(A^t A)$. In this work we used $\lambda = 0.1$.

Compressed Sensing

The CS theory originates from the field of high-dimensional statistics [3], and introduces a novel sampling process that goes against the common wisdom in data acquisition, i.e., Shannon-Nyquist Sampling theorem. CS theory asserts that one can recover certain signals from far fewer samples or measurements than traditional methods use [4].

Let f be the N-dimensional signal that we wish to measure/sample. Now let y be a sampled vector from f with length $\mathrm{M} < \mathrm{N}$. We can express the relationship between the signal and the sampled vector as $\mathrm{y} = \Phi\mathrm{f}$, where Φ is an $\mathrm{M} \times \mathrm{N}$ sampling matrix (the matrix that performs the projection from one space to another).

Furthermore, assume that the given signal can be represented by a basis set according to the equation $\mathrm{f} = \Psi\mathrm{x}$, where Ψ is a $\mathrm{N} \times \mathrm{N}$ matrix whose columns are the components of the basis set, and x is the $\mathrm{N} \times 1$ vector which contains the coefficients that analyze the signal f in basis Ψ.

According to the CS theory, the encoding of a given signal is a simple, linear sampling/measurement process whereby $\mathrm{y} = \mathrm{Ax}$ and $\mathrm{A} = \Phi\Psi$ (actualy equations $\mathrm{f} = \Psi\mathrm{x}$ and $\mathrm{y} = \Phi\mathrm{f}$ are combined). Matrix A is frequently referred as the measurement or the sensing matrix. Thus, having determined the x vector according to basis Ψ, we can extract a measurement/sampling vector by a mere matrix multiplication.

One crucial result of CS is that with overwhelming probability, by random measurements, e.g., i.i.d. entries from a symmetric Bernoulli distribution, only:

$$M \geq C \cdot a \cdot log(N/a) \tag{S14}$$

samples are needed for efficient reconstruction (C is an application specific constant and a is the sparsity measure of vector x). It can be proved that randomized sampling along with an l_1 minimization process as a decoding procedure, i.e., estimation of vector x by using measurements y, constitute a near-optimal sensing strategy [4]. Thus, in this work we used S14 for the determination of the size of the y vector with $C = 1$.

References

1. Selesnick IW (2010) Sparse Signal Restoration: 1–16. Available: http://eeweb.poly.edu/iselesni/teaching/lecture_notes/sparse_signal_restoration.pdf.

2. Figueiredo MAT, Bioucas-Dias JM, Nowak RD (2007) Majorization–Minimization Algorithms for Wavelet-Based Image Restoration. IEEE Trans Image Process 16: 2980–2991. Available: http://www.ncbi.nlm.nih.gov/pubmed/18092597. Accessed 4 June 2014.

3. Baraniuk RG (2011) More is less: signal processing and the data deluge. Science 331: 717–719. Available: http://www.ncbi.nlm.nih.gov/pubmed/21311012. Accessed 24 March 2014.

4. Candes EJ, Wakin M (2008) An Introduction To Compressive Sampling. IEEE Signal Process Mag 25: 21–30. Available: http://ieeexplore.ieee.org/xpls/abs_all.jsp?arnumber=4472240. Accessed 26 May 2014.
